# Sex differences in basal hypothalamic anorectic and orexigenic gene expression and the effect of quantitative and qualitative food restriction

**DOI:** 10.1186/s13293-018-0178-6

**Published:** 2018-05-29

**Authors:** S. D. Caughey, P. W. Wilson, N. Mukhtar, S. Brocklehurst, A. Reid, R. B. D’Eath, T. Boswell, I. C. Dunn

**Affiliations:** 1 The Roslin Institute and Royal (Dick) School of Veterinary Studies, University of Edinburgh, Easter Bush, Midlothian, Edinburgh, EH25 9RG Scotland, UK; 2 Bioinformatics and Statistics Scotland, Edinburgh, Scotland, UK; 3 Scotland’s Rural College, Edinburgh, Scotland, UK; 4 School of Natural and Environmental Sciences, Newcastle University, Newcastle upon Tyne, England, UK

**Keywords:** Sex, Psyllium, AGRP, POMC, Satiety, Growth, Body weight

## Abstract

**Background:**

Research into energy balance and growth has infrequently considered genetic sex, yet there is sexual dimorphism for growth across the animal kingdom. We test the hypothesis that in the chicken, there is a sex difference in arcuate nucleus neuropeptide gene expression, since previous research indicates hypothalamic *AGRP* expression is correlated with growth potential and that males grow faster than females. Because growth has been heavily selected in some chicken lines, food restriction is necessary to improve reproductive performance and welfare, but this increases hunger. Dietary dilution has been proposed to ameliorate this undesirable effect. We aimed to distinguish the effects of gut fullness from nutritional feedback on hypothalamic gene expression and its interaction with sex.

**Methods:**

Twelve-week-old male and female fast-growing chickens were either released from restriction and fed ad libitum or a restricted diet plus 15% *w*/*w* ispaghula husk, a non-nutritive bulking agent, for 2 days. A control group remained on quantitative restriction. Hypothalamic arcuate nucleus neuropeptides were measured using real-time PCR. To confirm observed sex differences, the experiment was repeated using only ad libitum and restricted fed fast-growing chickens and in a genetically distinct breed of ad libitum fed male and female chickens. Linear mixed models (Genstat 18) were used for statistical analysis with transformation where appropriate.

**Results:**

There were pronounced sex differences: expression of the orexigenic genes *AGRP* (*P* < 0.001) and *NPY* (*P* < 0.002) was higher in males of the fast-growing strain. In genetically distinct chickens, males had higher *AGRP* mRNA (*P* = 0.002) expression than females, suggesting sex difference was not restricted to a fast-growing strain. *AGRP* (*P* < 0.001) expression was significantly decreased in ad libitum fed birds but was high and indistinguishable between birds on a quantitative versus qualitative restricted diet. Inversely, gene expression of the anorectic genes *POMC* and *CART* was significantly higher in ad libitum fed birds but no consistent sex differences were observed.

**Conclusion:**

Expression of orexigenic peptides in the avian hypothalamus are significantly different between sexes. This could be useful starting point of investigating further if AGRP is an indicator of growth potential. Results also demonstrate that gut fill alone does not reduce orexigenic gene expression.

**Electronic supplementary material:**

The online version of this article (10.1186/s13293-018-0178-6) contains supplementary material, which is available to authorized users.

## Background

Sexual dimorphism is all around us; differences in plumage, pelage or ornamentation are observed across the animal kingdom with many species also displaying difference in body size and weight [1]. Most commonly, males are larger than females, but in some cases, the female is the largest sex with many examples in predatory birds, but also cases in mammals such as the blue whale and spotted hyena [2–6]. Research into the role of gene products such as agouti-related protein (AGRP) and pro-opiomelanocortin (POMC) in the control of energy balance in birds, or in mammals, has not paid particular attention to the sex of the animals studied [7]. Given the large difference in growth between males and females of many species, this is surprising, although as in our own studies on food intake and metabolism, a focus on one sex is sometimes made for industrial relevance [8–10]. In many galliforms, as in most mammals, the male grows larger and faster than the female; indeed, the domestic chicken displays one of the clearest sexual dimorphisms in body weight, with males around 20% heavier and with a clear difference in growth rate long before sexual or somatic maturity [11, 12]. This dimorphism for body weight holds true across the spectrum of chicken lines: in fast-growing meat-type chickens, in crosses between O-Shamo game bird and white leghorns and in egg-laying strains [11, 13, 14]. Selection for growth appears not to have in any way altered the sex difference in body weight, and the genetic correlation between male and female sibs of meat-type chickens is extremely high, with no evidence that the sex difference between them has a heritable component [11]. In other words, the differences in body weight are related entirely to the sex chromosomes inherited (in birds male ZZ, female ZW), and not to any interaction with the rest of the genome. From a practical point of view, selecting either sex for improved growth rate would be equally effective.

The central regulation of energy balance is conserved between birds and mammals, with the arcuate nucleus of the hypothalamus containing one population of neurons producing both AGRP and neuropeptide Y (NPY) with another synthesising α-melanocyte-stimulating hormone (αMSH) and other peptides from the *POMC* gene, and co-expressing cocaine- and amphetamine-regulated transcript (*CART*) mRNA [15–17]. The balance of POMC and AGRP is critical for controlling food intake; POMC neurons produce αMSH which acts on melanocortin 4 receptors (MC4Rs) to inhibit food intake, and contrastingly, AGRP acts as an antagonist on the same receptors to increase food intake and energy storage [18, 19]. Intracerebroventricular (ICV) injection of AGRP attenuated the anorectic effect of αMSH on food intake in both layer and broiler chicks; however, only in layer chicks did AGRP increase food intake under ad libitum feeding conditions suggesting the orexigenic effects of AGRP are different between layers and broilers [19]. We have previously shown that the expression of *AGRP* mRNA in the arcuate nucleus is increased many-fold in broiler breeder chickens under feed restriction compared to those fed ad libitum, whereas the anorectic peptide *POMC* mRNA was relatively unchanged [10]. In a line of chickens segregating at the cholecystokinin A receptor (*CCKAR*) locus, the autosomal genomic locus with the largest effect on growth and body weight, hypothalamic *AGRP* expression was higher in the animals carrying the high growth allele which had lower *CCKAR* mRNA and protein expression [8]. Furthermore, hypothalamic *AGRP* expression in growing chickens was shown to be responsive to both short- and long-term food availability [10]. This indicated that the AGRP neurones have a potentially important role in the control of feeding behaviour in birds because the level of *AGRP* mRNA represented not only the immediate satiety state of the bird but also how far the bird was from its body weight if it had not been food restricted [10]. In other words, hypothalamic *AGRP* expression in a number of different situations appears consistent in giving an indication of growth potential as much as short-term motivation to eat. Therefore, as our primary aim, we wanted to determine if genetic sex would be reflected in differences in *AGRP* expression.

There is currently a great deal of interest in the control of food intake in the context of overconsumption and obesity in humans whilst in domesticated animals it is particularly important for the efficient growth and production of meat. Chicken meat and eggs provide at least a third of the world’s animal protein [20]. Genetic selection in meat-type chickens has led to threefold increases in growth and feed efficiency [21]. Capitalising on this genetic potential has come with some adverse consequences. The parents of these meat-type chickens, known as broiler breeders, become overweight if allowed to feed ad libitum during rearing and to a lesser extent through the reproductive period, leading to poor welfare, decreased productivity and increased morbidity and mortality of up to 31% [22–26]. Food restriction is used successfully in the poultry industry to control these issues, with peak restriction around 25% of the ad libitum intake at 7–14 weeks of age. The birds show high levels of food motivation, with broiler breeders willing to experience an aversive stimulus to perform exploratory and foraging behaviour even when there was no food reward [27]. These conflicting welfare issues became known as the ‘broiler breeder paradox’ [24, 28, 29]. Understanding how growth is controlled and how differences in growth are genetically determined are, therefore, of key interest. Furthermore, investigating how the activity of anorectic and orexigenic neurons is related to growth is of potential importance in allowing it to be manipulated. One potential solution to the welfare problem of food-restricted broiler breeders experiencing prolonged hunger is to move from quantitative restriction to qualitative restriction by using dietary diluents to effectively lengthen the feeding period, change behaviour and apparently increase satiation whilst still restricting body weight [30–34]. The question remains, however, as to whether these approaches increase satiation indicators centrally. A second objective of this study was therefore to investigate whether the short-term inclusion of a fibrous bulking agent, the arabinoxylan fibre source Psyllium, also known as ispaghula husk, in the diet alters the gene expression of arcuate nucleus neuropeptides in a release from restriction model [10]. Psyllium in humans and laboratory rodents has been shown to have satiating effects, attributed to the effects of slowing down the absorption of nutrients by increasing bulk through its action of absorbing of large amounts of water. Psyllium is also not digestible or fully fermentable due to its complex polysaccharide structure, but it possibly increases the production of short-chain fatty acids in the distal gastrointestinal tract which may have satiating effects [35–37].

This study therefore had two main aims: firstly, to test the hypothesis that there is a sex difference in gene expression of neuropeptides controlling food intake in the hypothalamic feeding circuitry. Secondly, to test the hypothesis that qualitative food restriction induces a different pattern of gene expression in the arcuate nucleus compared to quantitative restriction; in other words, to distinguish the effects of gut fullness from nutritional signal feedback on the expression of central orexigenic and anorexigenic signals. These two aims were examined together to determine if there was any interaction between sex and dietary restriction, whether qualitative or quantitative, on hypothalamic gene expression.

## Methods

### Animal experiments

#### Sex and diet effect: To test the effect of sex and release from quantitative food restriction to qualitative restriction on basal hypothalamic neuropeptide gene expression

Un-sexed mixed female and male Ross 308 broiler breeders were group housed in three batches (hatches, *n* = 24) from hatch until 1 week prior to the experiment. Lighting, nutritional composition of the food and dietary restriction from day-old to 11 weeks of age was implemented in accordance with the breeders’ 2016 management manual (http://eu.aviagen.com/assets/Tech_Center/Ross_PS//308SF-PS-EU-PO-EN-16.pdf) and similar to detailed previously [10]. Our experiment was conducted when birds were 12 weeks of age which is within the peak period of food restriction for broiler breeders (7–14 weeks of age), a point where growth is almost at maximum and well before sexual maturity which typically occurs at around 20 weeks onward [38].

One week prior to the experiment, birds were weighed, ranked and randomised according to body weight and then assigned an individual cage and one of three treatment groups. For each of three replicate batches, six birds (*n* = 2 per treatment group) were transferred to their allocated individual cages in a new room on 4 successive days (different room for each day) and allowed to acclimatise for 6 days with continued commercial feed restriction. Birds were either then released from restriction and allowed to feed ad libitum (AL), fed the commercial restricted diet ration plus 15% *w*/*w* ispaghula husk (IH) or maintained on the commercial restricted diet ration (FR). The diets were fed for 2.5 days, and the birds were then killed with an intravenous injection of sodium pentobarbitol. In each batch, dissections (*n* = 6 per day) were performed when the birds were on average 11 weeks old over 4 days with equal numbers from the different treatment groups each day. All dissections were performed after 14.00 (7 h after lights on) with one bird from each triplet of treatment being sampled sequentially but randomly to minimise the effect of sample time. Basal hypothalamic brain tissue (40–100 mg) was dissected as previously described [39] and snap frozen on dry ice before being stored at − 80 °C until processed to extract RNA. The group size for each treatment was 24. Sex was determined at dissection with broadly equal number of each sex in each treatment. Ten birds (of 72) were lost to the study (2 AL, 3 FR and 3 IH) due to early illness.

#### Sex effect repeat: To repeat the experiment on the effect of sex and release from quantitative food restriction on the expression of anorectic and orexigenic peptide genes in the basal hypothalamus

Experiment section “Sex and diet” was repeated using the same line of chickens with the omission of the IH group to ensure the sex difference results observed in section “Sex and diet” were repeatable. Two replicate batches were used with dissections of eight birds (*n* = 4 per treatment group) performed when the birds were on average 12 weeks old over 2 days in each batch. The group size for each treatment was 16, designed with equal numbers of males and females per treatment based on genetic sexing [40]. One bird was lost to the study (AL female) and one AL bird thought initially to be female was male.

#### Genetically distinct line: To test the effect of sex on basal hypothalamic anorectic and orexigenic gene expression in a genetically distinct line of chickens

Female (*n* = 15) and male (*n* = 14) birds from the 20th generation of a broiler layer hybrid line [9] were reared in group housing under 14L:10D lighting and 26 °C temperature (ambient) and allowed to feed ad libitum on a standard grower diet until they were humanely killed at 10 weeks of age. There were five pens of bird used in the study which was confounded with hatch. All birds were heterozygotic for the previously described *CCKAR* locus alleles [9]. This line whilst not requiring feed restriction to maintain reproductive performance in adulthood does benefit from a moderate restriction that increases the production of viable eggs. Chickens were culled with an overdose of sodium pentobarbital and basal hypothalamus samples dissected as described for experiment section “Sex and diet effect.”

### Ethics statement

All animal experiments were performed under UK Home Office Project Licence 70/7909, and birds were humanely killed as specified in Schedule 1 of the UK Animals (Scientific Procedures) Act 1986.

### RNA extraction and reverse transcription

RNA was extracted from up to 100 mg of tissue with TRI-reagent (Ambion, Life Technologies, UK) and Lysing Matrix D tubes using a FastPrep Instrument FP120 (Thermo Electron Corporation, UK) and then purified according to manufacturers’ instructions using a Zymo Direct-zol™ RNA mini-prep kit (Cambridge BioSciences, UK). RNA concentration was read on a NanoDrop Spectrophotometer ND-1000 (LabTech International, UK). RNA (1 μg) was reverse transcribed using a high capacity cDNA reverse transcription kit (Applied Biosystems; Life Technologies, UK) following the manufacturer’s protocol before being diluted 5.5× and stored at − 20 °C.

### Real-time polymerase chain reaction (PCR) assays

Primers and assays were as described previously [10].

### Statistical analysis

All graphs and the table show means ± standard errors of means (SEMs) on the raw data scale, apart from expression measures that were standardised by dividing by the housekeeping gene. For experiment section “Genetically distinct line,” expression measures (log transformed) were analysed using an unbalanced ANOVA blocking for pen (identical to cull date) to investigate the effect of sex. Statistical analysis for experiment sections “Sex and diet effect” and Sex effect repeat were performed using linear mixed models (LMM) fitted to bird and organ weights (all log transformed except pituitary), crop content weight, feed intake (AL birds only) and expression measures (log transformed). In LMMs, random effects were included for batch (identical to the lab day for expression measures), the 12 (4 per batch) different days on which the birds were dissected (identical to spatial block), and individual birds (the residual). Body weight at post mortem was also investigated in the model, but this had little or no impact on differences between sexes in gene expression so is not reported here. Fixed effects were included for bird age (experiment section “Sex and diet effect” only, fitted as a four-level factor), diet treatment group (AL, FR, IH (experimental section “Sex and diet effect” only)), sex and the interaction between sex and treatment group. For experiment section “Sex and diet effect” because some of these are partially confounded, sequential tests were obtained testing these factors in four orders: age before and after treatment and sex, and treatment before and after sex. LMMs were fitted to all data and to data omitting outliers (as defined by the linear mixed model residuals) to confirm that results for all data reported here are not just attributable to the outliers. Post hoc tests were carried out by including contrasts in the fixed effects. *P* values reported here are the most conservative when alternative models were fitted. *P* values are based on approximate *F* tests when available but otherwise are based on Wald tests. Genstat (Genstat, 16th–18th editions, Lawes Agricultural Trust, VSN International Ltd.) was used for all statistical analyses.

## Results

### Effect of sex and diet on basal hypothalamic neuropeptide gene expression plus physiological parameters

#### Food intake and body and organ weight

Restricted birds were fed 46 g/day which equates to approximately 25% of the food intake of the average ad libitum intake (181 g/day) at 12 weeks of age, as previously observed [10]. Birds on the IH diet were fed the restricted diet containing 15% *w*/*w* ispaghula husk (52.9 g/day in total).

There was no difference in the food consumed in the days after release from restriction between males and females in the AL group (*P* = 0.792) in experiment section “Sex and diet effect”. Similarly, there was no significant difference in body weight between males and females overall (*P* = 0.787). In experiment section “Sex effect repeat” again no differences were observed in the food intake between males (186.33 ± 7.17 g) and females (188.0 ± 8.63 g) in the AL group after release from restriction (*P* = 0.885) and no significant difference in body weight between sexes overall (*P* = 0.801) nor was there an interaction with treatment was observed (*P* = 0.930; AL female 1795.5 ± 87.7 g, AL male 1785.3 ± 38.8 g, FR female 1515.1 ± 58.9 g, FR male 1495.0 ± 41.0 g).

As expected, average body weight varied with treatment group (*P* < 0.001, *F*_2,46_ = 127.14) with the AL group (1550.9 ± 28.1 g) average weight significantly higher (*P* < 0.001) than that for the FR (1229.0 ± 24.3 g) and IH (1225.9 ± 24.5 g) groups (*F*_1,46_ = 191.14, 187.30, respectively). There was no significant difference in the body weights between the FR and IH groups (*P* = 0.940). Average crop content weight varied with treatment (*P* < 0.001, *F*_2,46_ = 60.31) and was significantly higher in the IH fed group (56.14 ± 6.38 g) compared with the FR group (9.68 ± 2.43 g, *P* < 0.001, *F*_1,46_ = 48.38) but lower in the IH group than the AL group (83.02 ± 4.59 g, *P* < 0.001, *F*_1,46_ = 15.79). It was noted that whilst the restricted birds ate their ration in less than an hour, birds fed the restricted diet supplemented with IH took the entire daytime period of 14 h to finish the ration.

There were highly significant (*P* < 0.001, *F*_2,46_ = 87.68 pancreas, 75.64 liver, 47.20 gizzard, *F*_2,47_ = 47.78 proventriculus, *F*_2,42_ = 8.82 empty gall bladder) differences in organ weights (Table 1) between treatment groups in experiment section “Sex and diet effect” with the AL group having, on average, a significantly larger liver (*P* < 0.001, *F*_1,46_ = 80.37, 137.19), gizzard (*P* < 0.001, *F*_1,45_ = 23.88, *F*_1,46_ = 94.39), proventriculus (*P* < 0.001, *F*_1,46_ = 56.78, *F*_1,47_ = 83.34), empty gall bladder (*P* = 0.012, *F*_1,52_ = 6.78, *P* < 0.001, *F*_1,52_ = 17.19) and pancreas (*P* < 0.001, *F*_1,45_ = 148.06, *F*_1,46_ = 110.11) than the FR and IH groups. Interestingly, the FR group had significantly larger gizzards (*P* < 0.001, *F*_1,46_ = 21.93) and livers (*P* = 0.009, *F*_1,46_ = 7.38) than the IH group. In relation to sex, only the liver was significantly larger on average in the females compared to the males (*P* = 0.015, *F*_1,51_ = 6.30). There were no statistically significant differences in the weights of other organs between sexes or significant interactions between treatment and sex.

**Table 1:**
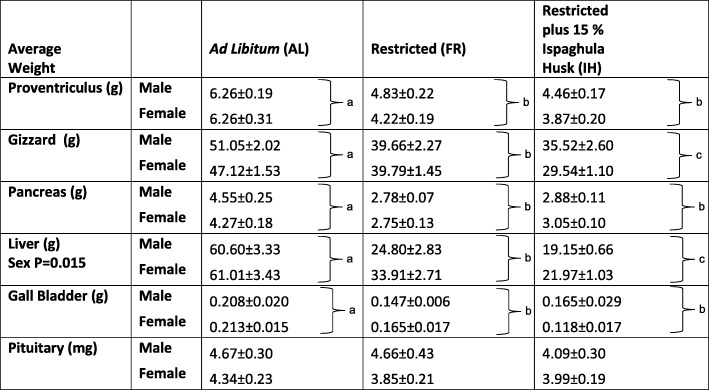
Whole organ weights (mean ± SEM) for broiler breeders after 2.5 days of a different feeding regime. Average organ weights for the ad libitum (AL; *n* = 21) fed group compared with the food restricted (FR; *n* = 20) and the food restricted plus 15% ispaghula husk (IH; *n* = 21) groups. *P* values are from LMMs with different labels (a, b, c) indicating differences between means from post hoc tests for the main treatment group effect

#### Effect of release from restriction to an ad libitum diet or diet containing a non-nutritious bulking agent on basal hypothalamic anorectic and orexigenic gene expression

**Fig. 1:**
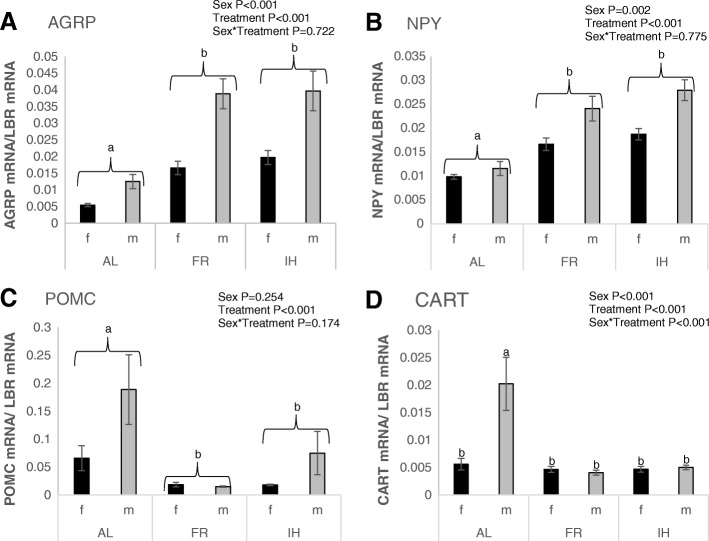
Gene expression (mean ± SEM) in the basal hypothalamus of male (m) and female (f) broiler breeders following different diets in experiment section “Sex and diet effect” Orexigenic (*AGRP* (**A**) and *NPY* (**B**)) and anorectic (*POMC* (**C**) and *CART* (**D**)) gene expression in birds fed ad libitum (AL; *n* = 19), feed restricted (FR; *n* = 20) and birds re-fed with ispaghula husk (IH; *n* = 21). *P* values are from LMMs with different labels (a, b) indicating statistically significant (*P* < 0.05) differences between means for the treatment group effect and for the sex by treatment group interaction

**Fig. 2:**
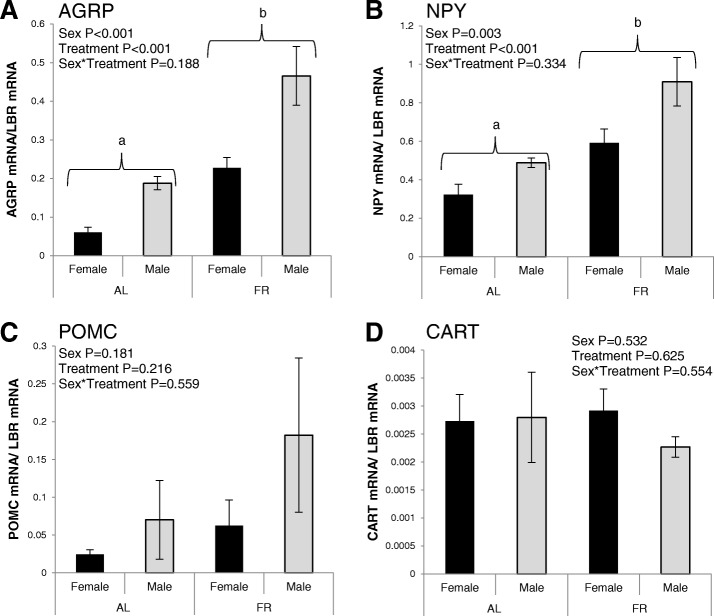
Anorectic and orexigenic gene expression (mean ± SEM) in the basal hypothalamus of male and female broiler breeders following release from commercial restriction in experiment section “Sex effect repeat” Expression of mRNA for *AGRP* (**A**), *NPY* (**B**), *POMC* (**C**) and *CART* (**D**) in the ad libitum (AL; *n* = 16) and commercial food-restricted (FR; *n* = 16) fed groups. *P* values are from LMMs with different labels (a, b) indicating statistically significant (*P* < 0.05) differences between means for the treatment group effect

In experiment section “Sex and diet effect” there was a highly significant (*P* < 0.001) difference between treatment groups in average expression of *AGRP*, *NPY*, *POMC* and *CART* (*F*_2,51_ = 50.45, 44.88, 12.11, 9.86, respectively) in the basal hypothalamus of birds (Fig. 1). Expression of *AGRP* and *NPY* mRNA in the basal hypothalamus of birds released from restriction and allowed to feed AL was significantly decreased (*P* < 0.001) compared with FR birds in experiment sections “Sex and diet**”** (Fig. 1A, B), (*AGRP F*_1,51_ = 70.43, *NPY F*_1,51_ = 50.54) and “Sex effect repeat” (Fig. 2A, B, *AGRP F*_1,25_ = 27.98, *NPY F*_1,25_ = 21.67). However, in the basal hypothalamus of birds fed the IH diet expression of *AGRP* (*P* < 0.001 vs AL, *F*_1,51_ = 81.69) and *NPY* (*P* < 0.001 vs AL, *F*_1,51_ = 81.34) mRNA was high and indistinguishable from that for FR birds (Fig. 1A, B). In experiment section “Sex and diet effect” an inverse pattern was observed for the anorectic genes, *POMC* and *CART*, with significantly higher expression seen in the AL birds compared to the FR (*POMC*, *P* < 0.001, *F*_1,51_ = 23.27; *CART*, *P* < 0.001, *F*_1,51_ = 17.61) and IH (*POMC*, *P* = 0.002, *F*_1,51_ = 11.09; *CART*, *P* = 0.001, *F*_1,51_ = 11.80) birds (Fig. 1C, D) and indistinguishable between IH and FR birds. In experiment section “Sex effect repeat” there was no significant effect of treatment groups on *POMC* (*P* = 0.216) and *CART* (*P* = 0.625) expression (Fig. 2C, D).

#### Sex differences in basal hypothalamic anorectic and orexigenic gene expression following release from restriction

A number of pronounced sex differences in gene expression in the basal hypothalamus were observed. Across the whole of experiment section “Sex and diet effect” the expression of *AGRP* (*P* < 0.001, *F*_1,51_ = 34.99) and *NPY* (*P* = 0.002, *F*_1,51_ = 11.13) mRNA was significantly higher in males compared with females (Fig. 1A, B). For *AGRP* and *NPY* mRNA, the sex difference in expression was larger within the restricted and IH re-fed groups than the AL group (Fig. 1A, B) although it should be noted that the interaction between sex and treatment was not significant for *AGRP* (*P* = 0.722) or *NPY* (*P* = 0.775).

For anorectic genes in the basal hypothalamus, the interaction between sex and treatment was highly significant (*P* < 0.001, *F*_2,51_ = 8.44) for *CART* mRNA expression which was significantly higher in the AL male group than in all other treatment by sex groups (*P* < 0.001, *F*_1,51_ = 22.71–32.91; Fig. 1A). There was no statistically significant effect of sex (*P* = 0.254) or sex by treatment (*P* = 0.174) on *POMC* mRNA expression in the basal hypothalamus.

The sex difference in expression of orexigenic genes in the basal hypothalamus was repeatable in experiment section “Sex effect repeat” (Fig. 2). In this experiment, *AGRP* (*P* < 0.001, *F*_1,25_ = 19.32) and *NPY* (*P* = 0.003, *F*_1,25_ = 10.71) mRNA expression was significantly higher in males compared to females (Fig. 2A, B). However, unlike in experiment section “Sex and diet effect” differences were not observed in the basal hypothalamic expression of anorectic genes between sexes (*POMC P* = 0.181, *CART P* = 0.532). There were no significant sex by treatment interactions observed in *AGRP* (Fig. 2A, *P* = 0.188), *NPY* (Fig. 2B, *P* = 0.334), *POMC* (Fig. 2C, *P* = 0.559), or *CART* (Fig. 2D, *P* = 0.554) mRNA expression in the repeat experiment.

### To test the effect of sex in basal hypothalamic anorectic and orexigenic gene expression in a genetically distinct chicken line

**Fig. 3:**
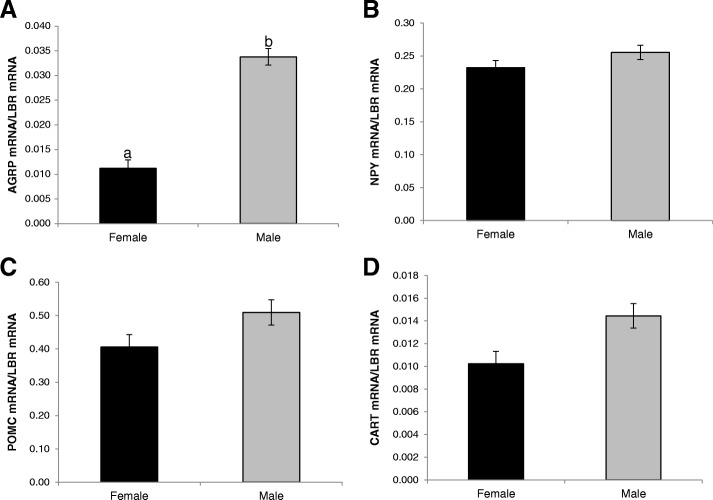
Anorectic and orexigenic gene expression (mean ± SEM) in the basal hypothalamus of male (*n* = 14) and female (*n* = 15) broiler layer hybrids (AIL) on an ad libitum diet. Expression of mRNA for *AGRP* (**A**), *NPY* (**B**), *POMC* (**C**) and *CART* (**D**). *P* values are from unbalanced ANOVA with different labels (a, b) indicating statistically significant (*P* < 0.05) differences between means for the effect of sex

In view of the dramatic sex differences observed in the broiler breeders, expression of arcuate nucleus genes were compared between males and females of an ad libitum fed advanced (20th generation) broiler layer hybrid line (AIL). Expression of *AGRP* mRNA was significantly higher in males compared with females (*P* = 0.002, *F*_1,23_ = 12.90) but *NPY* expression did not differ (*P* = 0.425; Fig. 3). For anorectic peptides, there was no difference in the mRNA expression of *CART* (*P* = 0.200) or *POMC* (*P* = 0.351) between sexes.

## Discussion

The observation of the dramatic differences in arcuate nucleus neuropeptide expression between sexes is novel and was initially surprising to us. This has not, as far as we know, been previously investigated or observed in birds and little researched even in mammals [41–43]. In our experiments, both *AGRP* and *NPY* expression were significantly higher in males than females. The sex differences in orexigenic peptide expression were repeatable and, for *AGRP*, also observed to be higher in males of a genetically distinct line of chickens. The observation of no difference in *NPY* gene expression in the genetically distinct line of chickens may be explained by the fact they were fed ad libitum for the whole experiment. Alternatively, because NPY is relatively ubiquitous in the brain and serves other functions in contrast to AGRP, which is confined to one group of neurons, the effect of sex on the *NPY* mRNA in the arcuate nucleus neurones may have been diluted.

These results support existing evidence that *AGRP* expression relates closely to the growth potential of the bird, as much as to its nutritional state. Our previous studies found that *AGRP* expression levels were significantly higher in birds released from restriction for 2 weeks compared with birds released for 6 weeks prior to cull, and those having been released longest had greater body mass [10]. Thus, *AGRP* mRNA expression was highest in birds furthest from their growth potential. In the present study, *AGRP* mRNA levels were higher in males who are known to grow faster and are larger than females and therefore would be furthest from their ideal body weight [11, 12]. Experiment sections “Sex and diet effect” and “Sex repeat effect” were performed with birds under restriction so no difference was observed in growth between males and females; however, in a subsequent generation of the genetically distinct line (used in experiment section “Genetically distinct line”), we have observed significant differences in the growth and weight between males and females on ad libitum feeding (see Additional file 1 Figure S1). Further evidence in the literature associating the level of *AGRP* expression with growth potential is the observation that in chickens segregating at the *CCKAR* locus, which is responsible for a 19% difference in body weight by 12 weeks of age, the birds homozygous for the high growth *CCKAR* allele had higher *AGRP* expression than those carrying the low growth *CCKAR* allele [9]. Thus, *AGRP* mRNA expression was higher in birds that grow larger compared to those with the low growth genotype.

One of the actions of AGRP is to increase food intake; however, there was no difference in the present study in the daily food intake between males and females when released from restriction. This may be because of the very short-term period of the release from restriction. Equally, this study was on broiler breeders and it has been previously observed that an ICV injection of AGRP did not stimulate food intake in broiler chicks under ad libitum feeding conditions whereas it did in layers [19]. The authors in that paper suggested the orexigenic effects of AGRP therefore may be different between the two breeds [19]. This is in agreement with the present study, where we did not observe higher food intake in males despite their higher *AGRP* expression compared with females when allowed to feed ad libitum. This raises the possibility that the actions of AGRP on food intake (in broilers at least) might be separate to its effects on growth potential, thus highlighting a key area of interest and investigation in the interaction of AGRP with the central melanocortin system and its impact upon energy balance and growth. Mouse models of obesity already suggest an involvement of AGRP in growth regulation through interaction with the melanocortin system especially the MC4R. Overexpression of *AGRP* in the mouse results in obesity and targeted inactivation of the MC4R causes obesity with features similar to the agouti obesity syndrome [44, 45]. More pronounced effects of the melanocortin system on growth have been observed in teleost fish, with the sexual dimorphic difference in growth in zebrafish reversed by overexpression of the agouti-signalling protein, a melanocortin receptor antagonist [43]. In this species, targeted prevention of the translation of *AGRP* mRNA resulted in decreased larval growth, an effect mediated by the MC4R because *MC4R* knockout teleosts were resistant to the growth-supressing effects of AGRP [46]. Furthermore, recently, it has been shown chicken MC4R*s* are equipotently activated by αMSH and adrenocorticotrophic hormone (ACTH) and this is heightened by the presence of melanocortin-2 receptor accessory protein 2 (MRAP2) whilst AGRP acts as an inverse agonist and antagonist on both MC4Rs and MC3Rs [47]. This along with the co-expression of *MC4R*, *MC3R*, *AGRP*, *POMC* and *MRAP2* mRNAs in the chicken hypothalamus indicates they may be important in the control of energy balance in the chicken with similar mechanisms of action as observed in mammals and teleosts [47]. The evolutionary conserved actions of AGRP and the melanocortin system on energy balance across vertebrates leads us to hypothesise that AGRP neurons may be an integrative centre for the expression of genetic effects on growth potential.

We observed that *POMC* and *CART* expression were higher in males compared with females in the AL fed groups. There has been some discrepancy in the literature regarding *POMC* mRNA expression after re-feeding with examples of both increased expression and no effect [48, 49]. It appears that studies of males did see a difference between restricted and AL fed groups but studies of females did not, which matches the results observed in this study [49, 50]. Although it should be noted that these sex differences in anorectic neuropeptide expression were not observed in our repeat study, the experiment sample size was based on a power analysis for *AGRP* expression changes and the statistical power may not have been sufficient to detect changes in the expression of the *POMC* and *CART* genes.

In birds, it has been observed that male and female somatic cells in gynandromorphs respond in different ways to a common steroidal milieu with male and female characteristics developing according to the proportion of the respective male and female cells in a tissue [51–53]. This is known as ‘cell autonomous sexual identity’ (CASI) which suggests that cells appear to ‘know’ their sex and develop according to the genetic sex. This CASI theory could be used to explain the sexually dimorphic phenotypes observed in birds, but is still controversial as the evidence from gynandromorphs is mainly correlational. Yet, there is support for the CASI theory because the transplantation of cells from a host prior to gonad differentiation will go onto develop a somatic cell fate but maintain the differentiated gene expression of the sex of the donor tissue [51]. However, there is still a body of evidence that some of these sex chromosome-determined phenotypes are also influenced by gonadal hormones; one of the clearest examples being the chicken’s comb and wattle [54, 55]. In terms of growth in chickens, research has only been able to investigate the influence of gonadal hormones and observed that blocking aromatase with Fadrozole in the embryo, so effectively sex reversing females so they experience male hormone milieu but are genetically female, increased growth by day 42 post hatch and apparently attenuated the difference between males and females [56]. Also, body weight gain was inhibited by the application of androgens in male, female or castrated chickens [57]. In mice which have been engineered to have male (XY) or female (XX) chromosome complements but develop within each genotype to have male or female gonads, there are examples of genetic effects on body weight-related traits. Mice carrying female sex chromosomes, irrespective of gonadal type, demonstrated greater food intake during daylight hours and double the amount of adipose tissue [58]. Evidence of genes responsible for the genetic effect on gene expression and potential sex-linked genes responsible for the trans effects on those genes were identified [58].

It is clear that there are many interesting questions surrounding what, sex chromosomes or gonadal hormones, determines sex differences in the expression of the arcuate nucleus neuropeptide genes and potentially growth and body weight. Further research will be needed to explore these questions.

The addition of the bulking agent, ispaghula husk, had no significant effect on the expression of the orexigenic peptides in the basal hypothalamus compared to normal feed restriction; both groups had significantly higher *AGRP* and *NPY* expression than the AL group released from the restricted diet. This strongly suggests that the bulking agent supplies no mechanosensory signals from the gut to the brain to reduce expression of *NPY* and *AGRP* in the orexigenic neurons. These results are in line with other studies in which it was observed that the level of crop fill, used as a marker of recent food ingestion, was not associated with hypothalamic expression of *AGRP*, *POMC* or *NPY* [59]. Ispaghula husk was added to the diet as a non-nutritive bulking agent, and our visual observation that it appeared to physically fill the crop and gut suggested that it acted in this manner. Its effects are likely to be comparable to other diet bulking agents such as oat hulls where improvements in behaviour are observed whilst still limiting growth rate [60]. The AL group had a significantly increased body weight compared to the FR and IH group. This increase is unlikely to be entirely attributable to crop content weight as the IH group also had significantly higher values, but there was no difference in their body weights compared with the FR group. This suggested that the IH diet was nutritionally limited as expected. Further support for this is the absence of significant differences in expected direction (i.e. increasing in size) in the majority of organ weights between the FR and IH groups compared to the significantly increased values in the AL group. For example, after 2.5 days, the liver was more than double the weight in the AL group compared to the FR and IH groups (see Table 1 for other organ weights). These points together prove that the addition of ispaghula husk provided bulk but little or no nutrients; indeed, it was observed the IH group took most of the daylight period to eat their ration whereas the FR group food was eaten within the first 10 min, suggesting the bulking effect caused gut fill and slowed down food intake and passage. This is largely the mechanism put forward for its benefit in treating gastrointestinal mobility issues in humans [61–63].

In mammals, the vagal afferents are suggested to relay mechanoreceptive stimuli to the *nucleus tractus solitarius* in the hind brain and this signalling can be modified by the action of classic satiety peptides [64]. These afferents and the pathways they activate in the brain represent an important target for future studies to understand the control of food intake in birds. The role of mechanoreceptors in the gut and the afferent pathways to the brain may be more important in birds, but relatively little is known of their methods of action in birds at present [65, 66]. From what we have observed in these experiments, IH provided bulk and slowed food intake but resulted in no alteration in hypothalamic gene expression compared with restricted birds. It seems likely that the inhibitory effect of gut fill on food intake is achieved by a mechanism independent of an action on AGRP*/*NPY and POMC*/*CART neurons. This may be the case only in broiler breeders; as was discussed above, injection of AGRP had no effect on food intake but did increase it in layer chicks [19]. It may be that in broilers in particular, AGRP neuronal activity may actually relate to the state of energy balance and a bird’s growth potential as much as to predicting actual intake of food, particularly when there is no food choice as we have seen in a number of paradigms including the present study [9].

## Conclusion

To conclude, we observed clear sex differences in the expression of central food regulatory genes, which exist in genetically distinct lines of chickens and are independent of feeding status. The higher level of *AGRP* mRNA in males adds further evidence to the idea that the level of its expression in the chicken hypothalamus is an indicator of a bird’s growth potential. Although research into understanding how AGRP may determine growth is just at its beginning, the role of AGRP in modulating the central melanocortin system is a good candidate mechanism for determining long-term growth as well as short-term food intake. Although IH bulked out the diet and spread food intake over a longer period, there were no changes in arcuate nucleus neuropeptide gene expression suggesting that mechanosensory signals do not impact upon the expression of satiety signals in the brain. However, the use of bulking agents to increase time spent showing feeding behaviour without metabolic satiation but whilst restricting growth seems to have welfare advantages from previous studies [30–34]. The mechanism underlying the apparent welfare improvement currently remains unknown but may involve neural signals from the gut to the brain via vagal afferents.

## Additional file

Additional file 1: **Figure S1.** Average weekly body weights for male and female chickens in a genetically distinct line. The average weekly body weights for male (n=57) and female (n=52) from broiler layer hybrid line fed on an *ad libitum* diet. P values are from a repeated measure ANOVA with different labels (*=*p*<0.05, **p<0.01, ***p<0.001) indicating differences between means from post hoc tests. (PDF 361 kb)

## Abbreviations

ACTH: Adrenocorticotrophic hormone
AGRP: Agouti-related peptide
AL: Ad libitum diet
ANOVA: Analysis of variance
CART: Cocaine- and amphetamine-regulated transcript
CASI: Cell autonomous sexual identity
CCKAR: Cholecystokinin A receptor
FR: Commercial restricted diet
ICV: Intracerebroventricular
IH: Commercial restricted diet plus 15% *w*/*w* ispaghula husk
MC4R(s): Melanocortin 4 receptor(s)
NPY: Neuropeptide Y
PCR: Polymerase chain reaction
SEM: Standard error of the mean
αMSH: Α-melanocyte-stimulating hormone
