# Supplementary material for: Sex differences in basal hypothalamic anorectic and orexigenic gene expression and the effect of quantitative and qualitative food restriction
Source: Biol Sex Differ. 2018 May 29;9:20. doi: 10.1186/s13293-018-0178-6 (PMC5975468; doi:10.1186/s13293-018-0178-6)
Supplement: Supplementary file 1 — Figure S1. Average weekly body weights for male and female chickens in a genetically distinct line. The average weekly body weights for male (n=57) and female (n=52) from broiler layer hybrid line fed on an ad libitum diet. P values are from a repeated measure ANOVA with different labels (*=p<0.05, **p<0.01, ***p<0.001) indicating differences between means from post hoc tests. (PDF 361 kb) [file 13293_2018_178_MOESM1_ESM.pdf]

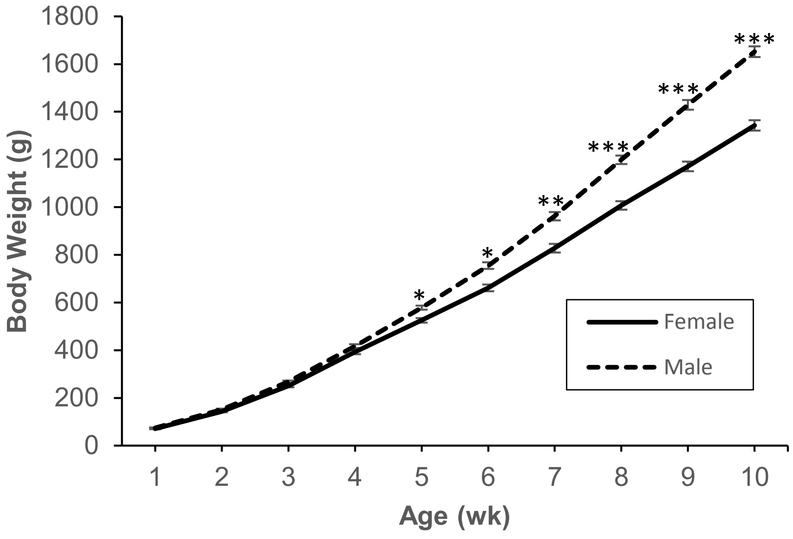

Supplementary Data Figure 1: Average weekly body weights for male and female chickens in a genetically distinct line. The average weekly body weights for male (n=57) and female (n=52) from broiler-layer hybrid line fed on an *ad libitum* diet. P values are from a repeated measure ANOVA with different labels (*=p<0.05, **p<0.01, ***p<0.001) indicating differences between means from post hoc tests.
